# Machine learning algorithms assisted identification of post-stroke depression associated biological features

**DOI:** 10.3389/fnins.2023.1146620

**Published:** 2023-03-08

**Authors:** Xintong Zhang, Xiangyu Wang, Shuwei Wang, Yingjie Zhang, Zeyu Wang, Qingyan Yang, Song Wang, Risheng Cao, Binbin Yu, Yu Zheng, Yini Dang

**Affiliations:** ^1^Department of Rehabilitation Medicine, The First Affiliated Hospital of Nanjing Medical University, Nanjing, Jiangsu, China; ^2^Department of Rehabilitation Medicine, The Affiliated Lianyungang Oriental Hospital of Kangda College of Nanjing Medical University, Lianyungang, Jiangsu, China; ^3^Department of Critical Care Medicine, Taizhou Hospital of Zhejiang Province Affiliated to Wenzhou Medical University, Taizhou, Zhejiang, China; ^4^Department of Rehabilitation Medicine, Shanghai Ruijin Rehabilitation Hospital, Shanghai, China; ^5^Department of Neurological Rehabilitation, Wuxi Yihe Rehabilitation Hospital, Wuxi, Jiangsu, China; ^6^Department of Science and Technology, The First Affiliated Hospital of Nanjing Medical University, Nanjing, Jiangsu, China; ^7^Department of Gastroenterology, The First Affiliated Hospital of Nanjing Medical University, Nanjing, Jiangsu, China

**Keywords:** post-stroke depression, metabolism, WGCNA, machine learning algorithms, GEO

## Abstract

**Objectives:**

Post-stroke depression (PSD) is a common and serious psychiatric complication which hinders functional recovery and social participation of stroke patients. Stroke is characterized by dynamic changes in metabolism and hemodynamics, however, there is still a lack of metabolism-associated effective and reliable diagnostic markers and therapeutic targets for PSD. Our study was dedicated to the discovery of metabolism related diagnostic and therapeutic biomarkers for PSD.

**Methods:**

Expression profiles of GSE140275, GSE122709, and GSE180470 were obtained from GEO database. Differentially expressed genes (DEGs) were detected in GSE140275 and GSE122709. Functional enrichment analysis was performed for DEGs in GSE140275. Weighted gene co-expression network analysis (WGCNA) was constructed in GSE122709 to identify key module genes. Moreover, correlation analysis was performed to obtain metabolism related genes. Interaction analysis of key module genes, metabolism related genes, and DEGs in GSE122709 was performed to obtain candidate hub genes. Two machine learning algorithms, least absolute shrinkage and selection operator (LASSO) and random forest, were used to identify signature genes. Expression of signature genes was validated in GSE140275, GSE122709, and GSE180470. Gene set enrichment analysis (GSEA) was applied on signature genes. Based on signature genes, a nomogram model was constructed in our PSD cohort (27 PSD patients vs. 54 controls). ROC curves were performed for the estimation of its diagnostic value. Finally, correlation analysis between expression of signature genes and several clinical traits was performed.

**Results:**

Functional enrichment analysis indicated that DEGs in GSE140275 enriched in metabolism pathway. A total of 8,188 metabolism associated genes were identified by correlation analysis. WGCNA analysis was constructed to obtain 3,471 key module genes. A total of 557 candidate hub genes were identified by interaction analysis. Furthermore, two signature genes (SDHD and FERMT3) were selected using LASSO and random forest analysis. GSEA analysis found that two signature genes had major roles in depression. Subsequently, PSD cohort was collected for constructing a PSD diagnosis. Nomogram model showed good reliability and validity. AUC values of receiver operating characteristic (ROC) curve of SDHD and FERMT3 were 0.896 and 0.964. ROC curves showed that two signature genes played a significant role in diagnosis of PSD. Correlation analysis found that SDHD (*r* = 0.653, *P* < 0.001) and FERM3 (*r* = 0.728, *P* < 0.001) were positively related to the Hamilton Depression Rating Scale 17-item (HAMD) score.

**Conclusion:**

A total of 557 metabolism associated candidate hub genes were obtained by interaction with DEGs in GSE122709, key modules genes, and metabolism related genes. Based on machine learning algorithms, two signature genes (SDHD and FERMT3) were identified, they were proved to be valuable therapeutic and diagnostic biomarkers for PSD. Early diagnosis and prevention of PSD were made possible by our findings.

## Introduction

Stroke remains the second leading cause of death and may lead to long-term disability in adults (GBD 2019 Stroke Collaborators, 2021; Sun et al., 2021). After the acute stage, most of stroke patients suffer from physical and mental disabilities of varying degrees, including hemiplegia, reduced energy, and disturbed sleep (Zhang et al., 2013; Dong et al., 2021). Previous studies have shown that about 30–40% of stroke patients develop post-stroke depression (PSD) which is a mood disorder characterized by depression and anhedonia, and is associated with decreased rehabilitation motivation, reduced quality of life, poor functional outcome, as well as increased cost of treatment and burden of family caregiver (Li et al., 2020). One meta-analysis concluded that a hazard ratio for post-stroke depression and all-cause mortality was 1.59 (Cai et al., 2019). However, PSD is often concealed due to unrecognized depressive symptoms and their decreased willingness of treatment attendance (Klinedinst et al., 2012). Diagnosis of PSD is currently based on clinical symptoms, and there is no reliable objective parameter. Therefore, it is necessary to explore the new diagnostic and therapeutic biomarkers for PSD in subacute period of stroke.

There is accumulating evidence that PSD and metabolism are intimately related. Compared with non-PSD, stroke patients with PSD have higher glutamate levels in the frontal lobe (Wang et al., 2012). Previous studies found that a high level of homocysteine has been identified as the qualifiable risk factor for ischemic stroke, and elevated serum level of homocysteine is also significantly associated with depression (Li et al., 2017; Zhou et al., 2018). Jiang et al. (2021) demonstrated that gut microbiome may participate in the development of PSD, the discriminating fecal metabolites were mainly involved in lipid metabolism, amino acid metabolism, carbohydrate metabolism and nucleotide metabolism. These results indicated that metabolism plays an important role in the pathological process of PSD.

Recently with the assistant of advanced sequencing technologies and machine learning algorithms, intelligent hub gene and signaling pathway detection becomes realistic. Several studies based on weighted gene co-expression network analysis (WGCNA) have reported changes in relevant key pathways and differential expression of key related genes in post-stroke patients (Li et al., 2020; Wang et al., 2020; Lin et al., 2021). Furthermore, Liu et al. (2022) used WGCNA combined with the random forest model and the least absolute shrinkage and selection operator (LASSO) analysis to identify 10 key genes in patients with Alzheimer’s Disease. However, these techniques have not been widely applied in the investigation of metabolism biomarkers of PSD.

Upon the above concerns, this study employed multiple bioinformatic approaches to find possible biomarkers. Firstly, three gene expression profiles of stroke were obtained from GEO database. Differentially expressed genes (DEGs) were detected. WGCNA was constructed to identify disease related module genes. Then, correlation analysis was performed to obtain metabolism related genes. Interaction analysis was performed to obtain candidate hub genes. Subsequently, signature genes were identified by LASSO and random forest analysis. Gene set enrichment analysis was applied on signature genes. Finally, a diagnosis model was built in PSD cohort. In general, the findings of this research may assist in the diagnosis and treatment of PSD as well as increase our understanding of etiology of PSD.

## Materials and methods

### Data sources and processing

Three datasets (GSE140275, GSE122709, GSE180470) were downloaded from Gene Expression Omnibus (GEO).^1^ The GSE140275 dataset contained six patients, including three healthy controls (HC) and three stroke patients. The GSE122709 dataset included five HC and ten stroke patients. GSE180470 dataset included three HC and three stroke patients. Expressions of three datasets were all derived from human blood tissue. “Limma” and “edgeR” package in R software was used to investigate differentially expressed genes (DEGs) (Robinson et al., 2010; Ritchie et al., 2015), which was specified as “*P*-value < 0.05 and log_2_ (fold change) > 1 or log_2_ (fold change) < –1.” For visualization, the volcano plots were generated to show DEGs, while the top 25 upregulated and the top 25 downregulated DEGs were displayed by heatmaps.

### Functional enrichment analysis

Functional enrichment analysis was conducted to evaluate major biological attributes of DEGs, specifically including Gene Ontology (GO) and Kyoto Encyclopedia of Genes and Genomes (KEGG) pathway analysis using “ClusterProfiler” package in R software. Threshold was set at *P*-value < 0.05. GO categories comprised biological processes (BP), molecular functions (MF), and cellular components (CC) (Zhu et al., 2022).

### Weighted gene co-expression network analysis (WGCNA)

Based on scale-free topology criterion, co-expression network in GSE122709 dataset was constructed using “WGCNA” package in R software to identify co-expression gene modules (Langfelder and Horvath, 2008). Briefly, genes with read counts less than 10 and “NA” were filtered out, top 5,000 variant genes were selected. Pearson’s correlations between each gene pair were calculated to build an adjacency matrix. Afterward, a “soft” threshold power (β) was estimated according to the criteria of scale-free topology to construct a biologically important scale-free network. Dynamic Tree Cut algorithm was then used to identify gene modules (Lin et al., 2021). Module membership (MM) and gene significance (GS) were estimated to connect modules with clinical characteristics. Hub gene modules were designated as those with the highest Pearson module membership correlation and *P*-value < 0.05 (Liu et al., 2021).

### Screening for candidate hub genes

Based on R software, “WGCNA” package was used for correlation analysis for genes in GSE122709 and seven genes associated with metabolism with the following parameters: | R| > 0.5, *P* < 0.001. Then, we identified candidate hub genes by the intersection of DEGs, key module genes and metabolism related genes. Finally, results were visualized by Venn diagram *via* online tool Venny 2.1.0^2^ (He et al., 2021).

### Identification for signature genes in patients with stroke

We screened candidate hub genes by the intersection of DEGs, key module genes and metabolism related genes. Subsequently, two machine learning algorithms, least absolute shrinkage and selection operator (LASSO) and random forest, were used to identify hub gene. LASSO, a dimension reduction approach, shows superiority in evaluating high-dimensional data in comparison to regression analysis (Kang et al., 2021). The “glmnet” package was used to implement LASSO analysis with a turning/penalty parameter utilizing a 10-fold cross-validation. Furthermore, the “random forest” package was used for performing the random forest analysis which determined the optimal number of variables by computing average error rate of candidate hub genes (Mantero and Ishwaran, 2021). A random forest tree model was built and the importance scores of each candidate hub genes were identified. Genes with importance value >0.25 were determined. The intersection genes of LASSO and random forest analysis were used to pick signature genes of patients with stroke.

### Establishment of nomogram

The “rms” package was applied for incorporating signature genes to establish a nomogram. The “score” is the score of the relevant item below, and the “total score” is the sum of all the elements above. Calibration curves were used for assessing the predictive power of the model. Clinical usefulness of nomogram was evaluated by decision curve analysis, which determines clinical practicability of nomogram by quantifying the net benefits under different threshold probabilities in the validation set. Furthermore, we performed clinical impact curves to evaluate clinical utility of the model (Xu et al., 2021).

### Curve analysis of receiver operating characteristics (ROC)

The “pROC” package was applied to create Receiver Operating Characteristic (ROC) curves to determine the area under the curve (AUC) for screening signature genes and evaluating their diagnostic value (Robin et al., 2011). AUCs of 0.5–0.7 were considered with low diagnostic accuracy, 0.7–0.9 were considered with moderate accuracy, and >0.9 indicates high accuracy.

### Gene set enrichment analysis (GSEA)

To functionally investigate the biological significance of signature genes, GSEA (version 4.1.0) was performed in different subgroups. KEGG gene sets were chosen as the gene set database (Subramanian et al., 2005). Normalized enrichment score (NES) and false discovery rate (FDR) were used to determine if differences were statistically significant and cut-off values were FDR < 0.25, *P* < 0.05, and | NES| > 1.

### PSD validation cohort

This was a cohort study enrolled at the First Affiliated Hospital of Nanjing Medical University from September 2020 to April 2022. It was approved by the Committee of Institutional Ethics (Institutional Review Board, 2018-SR-339) and all participants provided written informed consent prior to participation. Patients eligible for inclusion in the study were: (1) aged older than 18 years; (2) diagnosed with ischemic stroke on brain MRI; (3) with stable vital signs (Luft et al., 2004; Upreti et al., 2019). Patients were excluded if (1) presence of severe cognitive impairment; (2) participated in other clinical trials within 6 months (Shi et al., 2021; Yu et al., 2022).

All participants underwent an initial clinical assessment, including the collection of clinical and demographic information. Depression symptoms in post-stroke patients were evaluated by the Hamilton Depression Rating Scale 17-item (HAMD) at 1 month after stroke by a trained neurologist (Lin et al., 2020; Qiao et al., 2020). A score of 0–7 was considered normal, while a HAMD score ≥8 is indicative of depression. Stroke severity was measured using the National Institute of Health Stroke Scale (NIHSS) (He et al., 2020). Modified Rankin Scale (mRS) was used to estimate the functional disability (Liu et al., 2018). Independence and level of activities of daily life (ADL) were evaluated with the Barthel index (Kamal et al., 2020). For research purposes, a blood sample (10 ml) was taken from each subject for further ELISA assessment when they completed the HAMD assessment.

### ELISA analysis

Concentration of signature genes in serum of stroke patients were measured using ELISA kit (antibodies-online, Philadelphia, PA, USA). Briefly, 100 μL standard or sample were added to each well and incubated for 90 min at 37^°^C. After washing two times, 100 μL Biotin-labeled antibody working solution was added and incubated for 60 min for 37^°^C. After plates were washed three times. A total of 100 μL SABC Working Solution was added and incubated for 30 min at 37^°^C. Subsequently, 90 μL TMB Substrate Solution was added and incubated 20 min at 37^°^C. After the incubation, 50 μL stop solution was added into each well to stop the reaction. Finally, Absorbance value at 450 nm was read immediately and calculation (Kaida et al., 2013; Zhou et al., 2015).

### Statistical analysis

All statistical analyses in our study were implemented using R software (version 4.1.2). The difference between the two groups was analyzed by Student’s *t*-test. The correlation between genes in GSE122709 and metabolism related genes was determined using Pearson’s correlation test. All statistical *P*-values were two-sided, and statistical significance was considered with *P*-value < 0.05.

## Results

**FIGURE 1:**
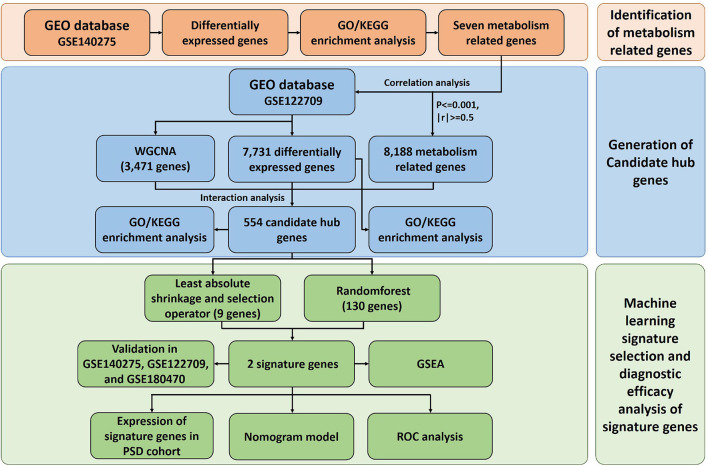
Flow chart. GEO, Gene Expression Omnibus; WGCNA, weighted gene co-expression network analysis; PSD, post-stroke depression; ROC, receiver operating characteristic; GO, gene ontology; KEGG, Kyoto Encyclopedia of Genes and Genomes; GSEA, gene set enrichment analysis.

Detailed procedure of our study is shown in Figure 1.

### Identification of DEGs between HC and stroke patients

**FIGURE 2:**
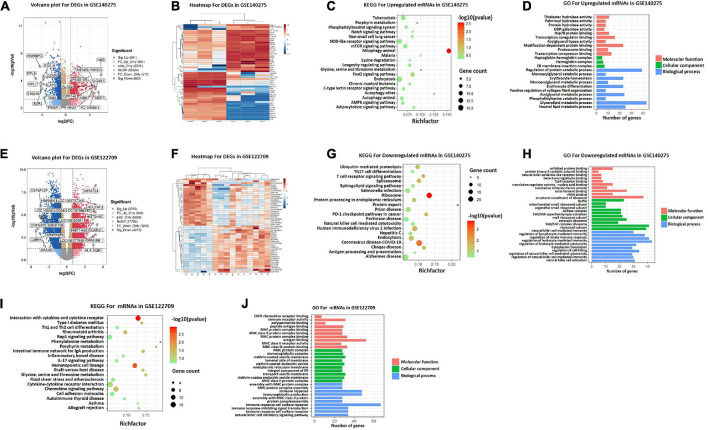
DEGs screening and functional enrichment analysis. **(A)** Volcano plot of differentially expressed genes in GSE140275. **(B)** Heatmap of differentially expressed genes in GSE140275. **(C)** KEGG pathway analyses of upregulated mRNAs in GSE140275. **(D)** GO functional analyses of upregulated mRNAs in GSE140275. **(E)** Volcano plot of differentially expressed genes in GSE122709. **(F)** Heatmap of differentially expressed genes in GSE122709. **(G)** KEGG pathway analyses of downregulated mRNAs in GSE140275. **(H)** GO functional analyses of downregulated mRNAs in GSE140275. **(I)** KEGG pathway analyses of mRNAs in GSE122709. **(J)** GO functional analyses of mRNAs in GSE122709. GO, Gene Ontology; KEGG, Kyoto Encyclopedia of Genes and Genomes; DEGs, differentially expressed genes.

To identify potential DEGs, expression profiles of GSE140275 and GSE122709 in GEO database were performed using “Limma” package with *P* < 0.05 and | logFC| > 1 as threshold. A total of 1,724 DEGs were screened in GSE140275 including 861 upregulated genes and 863 downregulated genes (Supplementary Table 1). A total of 7,731 DEGs were obtained, of which 3,516 genes presented upregulation and 4,215 genes presented downregulation in GSE122709 (Supplementary Table 2). The volcano plots were demonstrated in Figures 2A, E. The heatmap showed the top 25 upregulated and top 25 downregulated DEGs between healthy control and stroke patients (Figures 2B, F).

### Functional enrichment analysis of DEGs in GSE140275

Functional enrichment analysis was carried out to investigate the biological functions of DEGs in GSE140275. Among upregulated DEGs, KEGG enrichment analysis demonstrated that “autophagy,” “porphyrin metabolism,” and “glycine, serine and threonine metabolism” were highly enriched (Figure 2C); GO analysis showed that multiple metabolic pathways were also significantly enriched in biological processes, such as “monoacylglycerol metabolic process,” “acylglycerol metabolic process,” and “glycerolipid metabolic process” (Figure 2D). The results of KEGG showed downregulated DEGs were especially enriched in “ribosome,” “protein export,” and “T cell receptor signaling pathway” (Figure 2G). Additional GO analysis suggested downregulated DEGs were significantly enriched in “structural constituent of ribosome” in MFs, “ribosome” in CCs, and “regulation of leukocyte mediated immunity” in BPs (Figure 2H). Similarly, KEGG pathways analysis of GSE122709 showed that “porphyrin metabolism”, and “glycine, serine and threonine metabolism” were significantly enriched (Figures 2I, J), indicating that metabolism played an important role in stroke.

### Construction of the weighted gene co-expression network

**FIGURE 3:**
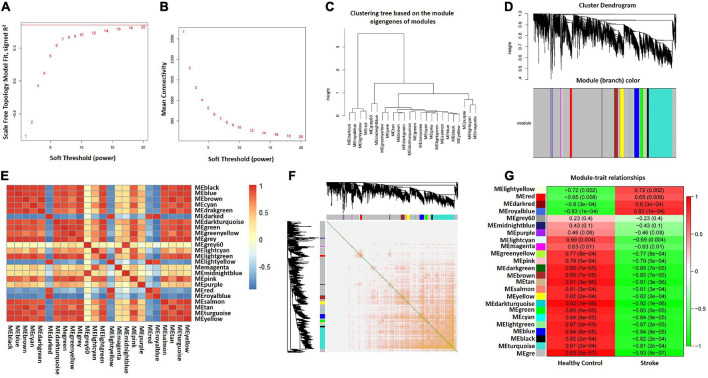
Construction of WGCNA co–expression network. **(A)** Scale-free fit index. **(B)** Mean connectivity. **(C)** Clustered dendrograms. **(D)** Clustering dendrogram of genes, various colors represent different modules. **(E)** Correlation heatmap between modules. Red and blue represent positive and negative correlations, respectively. **(F)** Clustering dendrogram of module feature genes. **(G)** Heatmap of module–trait correlations. Red and green represent positive and negative correlations, respectively. HC, healthy control; WGCNA, weighted gene co-expression network analysis.

The GSE122709 dataset (five HC and 10 stroke patients) was obtained for WGCNA analysis to identify modules of highly correlated genes. A scale-free co-expression network was constructed with the soft threshold to 20 and the mean connectivity is relatively favorable (Figures 3A, B). We selected 0.25 as clustering height limit to merge the strongly associated modules (Figure 3C). Subsequently, 24 signature modules were identified and labeled with different colors (Figure 3D). The correlation between modules was computed, and the results were showed in Figure 3E. In addition, transcription correlation analysis was performed and demonstrated that there was no substantive connection between modules (Figure 3F). Finally, we calculated the correlation between each module and clinical features. Results indicated that the MEroyalblue module was negatively correlated with HC (*r* = –0.83, *P* = 1e–04) and positively correlated with stroke (*r* = 0.83, *P* = 1e–04), while the Megrey module was negatively correlated with stroke (*r* = –0.93, *P* = 5e–07) and positively correlated with healthy control (*r* = 0.93, *P* = 5e–07) (Figure 3G and Supplementary Table 3). Therefore, Meroyalblue and Megrey modules were identified as clinically meaningful modules.

### Identification of metabolism related candidate hub genes

**FIGURE 4:**
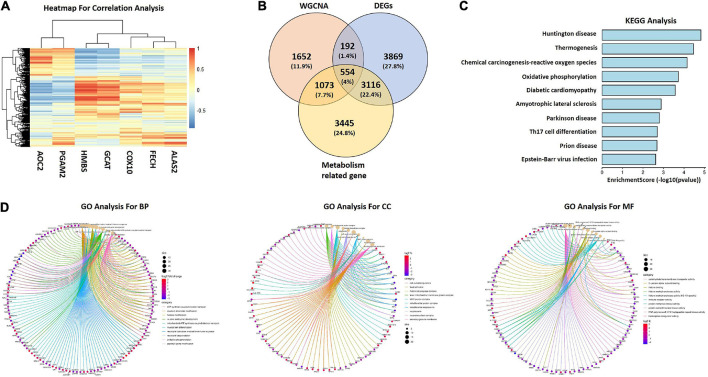
Generation of metabolism related candidate hub genes. **(A)** Correlation heatmap between seven metabolism related genes and DEGs in GSE122709. Red represents positive correlations, and blue represents negative correlations. **(B)** Venn diagram to identify candidate hub genes between metabolism related genes, key modules genes and DEGs. **(C)** KEGG analysis of candidate hub genes. **(D)** GO analysis of candidate hub genes. WGCNA, weighted gene co-expression network analysis; DEGs, differentially expressed genes; KEGG, Kyoto Encyclopedia of Genes and Genomes; GO, Gene Ontology; BP, biological processes; CC, cellular components; MF, molecular functions.

Based on KEGG pathway analysis in GSE140275, we extracted porphyrin metabolism and glycine, serine and threonine metabolism related genes (ALAS2, FECH, COX10, GCAT, HMBS, PGAM2, and AOC2). Correlation analysis between seven genes and all genes in GSE122709 dataset was conducted. A total of 8,188 metabolism related genes were identified (| r| ≥ 0.5, *P* ≤ 0.001). The heatmap of correlation analysis were shown in Figure 4A. Subsequently, we interacted DEGs in GSE122709, genes in Meroyalblue and Megrey modules, and metabolism related genes, 554 common genes were obtained as metabolism related candidate hub genes (Figure 4B). Functional enrichment analysis revealed that metabolism related candidate hub genes were enriched in “oxidative phosphorylation,” “ATP synthesis coupled electron transport,” “cell-substrate junction,” and “carbohydrate transmembrane transporter activity” (Figures 4C, D).

### Selection of signature genes *via* machine learning algorithms

**FIGURE 5:**
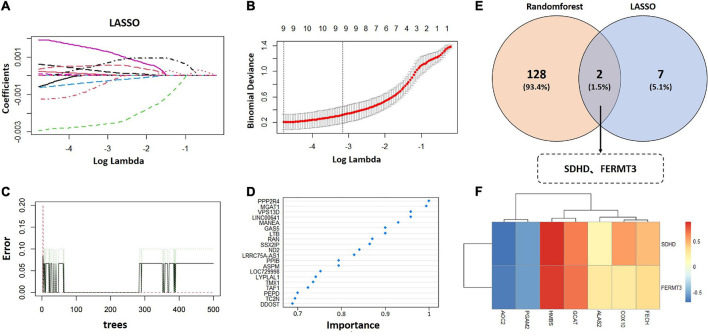
Selection of signature genes. **(A)** Parameter selection was performed through LASSO regression. **(B)** Elucidation of LASSO coefficient profiles for selected factors. **(C)** Random forest error rate versus the number of classification trees. **(D)** The top 20 relatively important genes. **(E)** Venn diagram to identify signature genes between LASSO and random forest. **(F)** Heatmap of correlation analysis between two signature genes and metabolism related genes (ALAS2, FECH, COX10, GCAT, HMBS, PGAM2, and AOC2). LASSO, the least absolute shrinkage and selection operator.

Least absolute shrinkage and selection operator and random forest algorithms were applied to identify signature genes from 554 metabolism related candidate hub genes. For LASSO analysis, nine signature genes were selected from statistically significant univariate variables (Figures 5A, B and Supplementary Table 4). For random forest analysis, we set importance value to 0.25 as the threshold and 130 signature genes were determined (Figures 5C, D and Supplementary Table 5). The interaction analysis of LASSO and random forest indicated that two signature genes were finally screened out, including succinate dehydrogenase complex subunit D (SDHD) and fermitin family member 3 (FERMT3) (Figure 5E). Finally, correlation analysis of two signature genes and metabolism related genes (ALAS2, FECH, COX10, GCAT, HMBS, PGAM2, and AOC2) demonstrated that SDHD and FERMT3 were significantly correlated with metabolism (Figure 5F).

### Validation of signature genes

**FIGURE 6:**
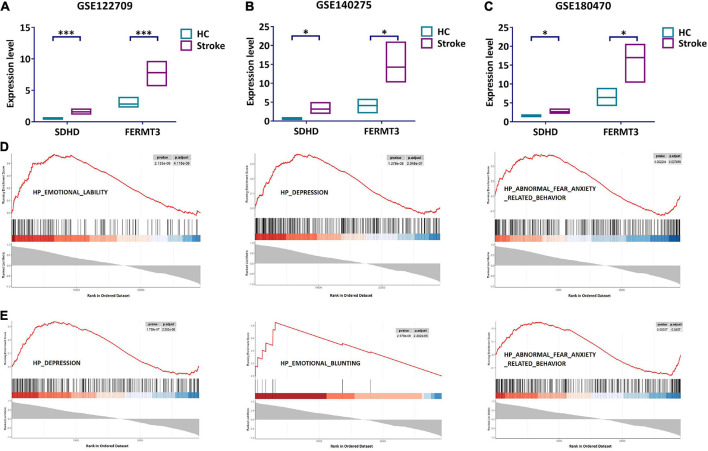
Validation and GSEA analysis of signature genes. **(A)** Expression level of SDHD and FERMT3 in GSE122709. **(B)** Expression level of SDHD and FERMT3 in GSE140275. **(C)** Expression level of SDHD and FERMT3 in GSE180470. **(D)** GSEA analysis of SDHD. **(E)** GSEA analysis of FERMT3. *P < 0.05; ^***^P < 0.001. HC, healthy control; GSEA, gene set enrichment analysis.

We further investigated the role of SDHD and FERMT3. The expression of SDHD and FERMT3 was verified in GSE140275 and GSE122709. The results showed that SDHD was substantially upregulated in the stroke group, while the same trend was seen in expression of FERMT3 (Figures 6A, B). To further confirm the reliability of our results, validation dataset (GSE180470) was used to validate expression of SDHD and FERMT3. SDHD and FERMT3 were highly expressed in the stroke group (Figure 6C), suggesting that these genes may play a significant role in stroke.

### GSEA analysis of signature genes

Gene set enrichment analysis was performed for evaluating signaling pathways involved in the signature genes. The results showed that SDHD was significantly correlated with “emotional lability,” “depression,” and “abnormal fear anxiety related behavior” (Figure 6D). Meanwhile, “depression,” “emotional blunting,” and “abnormal fear anxiety related behavior” were detected for FERMT3 (Figure 6E). The results indicated that SDHD and FERMT3 played a key role for diagnosis of psychosocial state in stroke patients.

### Diagnostic efficacy of signature genes in PSD patients

**FIGURE 7:**
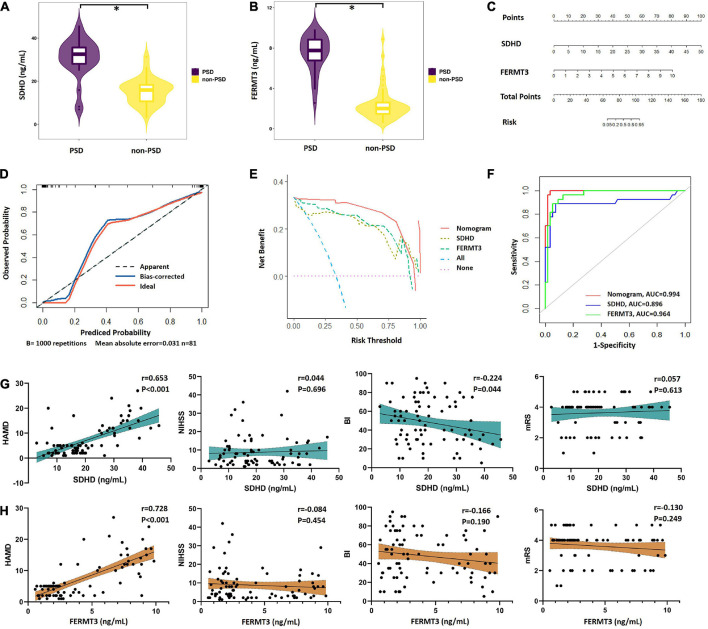
Performance of signature genes in PSD patients. **(A)** Expression level of SDHD in PSD and non-PSD groups. **(B)** Expression level of FERMT3 in PSD and non-PSD groups. **(C)** Nomograms for the prediction of the PSD risk. **(D)** Scatter diagram of calibration plot for internal verification of the nomogram model. **(F)** DCA curves of the nomogram model. **(E)** ROC curves of the nomogram model. **(G)** Correlation between expression of SDHD and four clinical traits (HAMD, NIHSS, BI, and mRS). **(H)** Correlation between expression of FERMT3 and four clinical traits (HAMD, NIHSS, BI, and mRS). PSD, post-stroke depression; DCA, decision curve analysis; ROC, receiver operating characteristic; AUC, area under the curve; HAMD, Hamilton Depression Rating Scale 17-item; NIHSS, National Institute of Health Stroke Scale; BI, Barthel index; mRS, Modified Rankin Scale. *P < 0.05.

Based on GSEA analysis of two signature genes (SDHD and FERMT3), we found that they have a significant correlation with depression. Therefore, we collected 81 stroke patients who were assigned into the PSD group (mean HAMD score = 14.74) and non-PSD group (mean HAMD score = 3.41). There was no difference in baseline clinical features between groups (Supplementary Table 6). Meanwhile, expression of serum SDHD and FERMT3 in all patients were detected by ELISA kit. SDHD and FERMT3 presented higher expression in the PSD group than the non-PSD group (Figures 7A, B), indicating their potential roles in diagnosis of depression in stroke patients. To predict diagnostic performance of signature genes in stroke patients with depression, the nomogram model for the signature genes (SDHD and FERMT3) was built using “rms” package (Figure 7C). Calibration curves demonstrated that the difference between the real and predicted depression risks was very minimal, indicating the nomogram model enabled an accurate estimation (Figure 7D). In addition, decision curves analysis demonstrated that the nomogram provided a greater clinical benefit (Figure 7E). The ROC curve also showed that the model was able to help clinicians accurately diagnose depression of stroke patients (Figure 7F). Additionally, correlation analysis between two signature genes and several clinical traits (HAMD, NIHSS, BI, and mRS) indicated that SDHD (*r* = 0.653, *P* < 0.001) and FERM3 (*r* = 0.728, *P* < 0.001) were positively related HAMD, while SDHD also displayed a negative association with Barthel index (*r* = –0.224, *P* = 0.044) (Figures 7G, H).

## Discussion

In this study, we included three datasets (GSE140275, GSE122709, GSE180470) with 27 patients for data analysis. We first screened 1,724 DEGs in GSE140275 including 861 upregulated genes and 863 downregulated genes. Subsequent KEGG enrichment analysis showed “porphyrin metabolism” and “glycine, serine and threonine metabolism” were highly enriched. Recent research reveals that stroke causes systemic complications, including hyperlipemia, high blood viscosity, dysfunctional gut microbiota, and a leaky gut (Yamashiro et al., 2017; Chen et al., 2019a). Chen et al. (2019b) demonstrated that stroke would cause gut microbiota dysbiosis, translocation of gut microbiota, and disruption to the gut barrier. And supplementation of short chain fatty acids (SCFAs), especially butyric acid, could remodel the gut microbiota and treat stroke (Chen et al., 2019a). Moreover, with the development of biology, metabolomics was applied to explore biomarkers and mechanisms of stroke by identifying metabolic alterations. Several studies reported the increase in ketone bodies levels in rats with stroke compared with sham group (Chen et al., 2019c; Wang et al., 2019). Fu et al. (2019) reported a decrease in β-hydroxybutyric acid level in serum but an increase in brain tissue in stroke rats, providing more energy for brain. These studies suggest that metabolism features strongly correlate with prevention, diagnosis and treatment of stroke. Based on the role of metabolism in stroke, we extracted seven genes related to the “porphyrin metabolism” and “glycine, serine and threonine metabolism” pathways, including ALAS2, FECH, COX10, GCAT, HMBS, PGAM2, and AOC2. We then performed correlation analysis between these genes enriched in these two pathways in GSE140275 and all genes in GSE122709 to identify metabolism related genes. A total of 8,188 metabolism related genes were identified. Nevertheless, with the help of advanced bioinformatic approaches genetic information could be further derived.

Weighted gene co-expression network analysis (WGCNA) is a frequently applied method to identify co-expression pattern at whole transcriptome level. Wang et al. (2019) performed WGCNA analysis to investigate co-expression modules related with osteosarcoma and found genes in brown module might be related with carcinogenesis of osteosarcoma. In addition, there were several studies screened key module genes related to stroke by WGCNA analysis (Fan et al., 2022; Zheng et al., 2022). However, metabolism related pathways and key genes in stroke are seldomly identified. Therefore, we performed WGCNA analysis of GSE122709 to identify 24 gene modules. No significant correlation between dividing modules was found. Module-traits relationship analysis indicated that Meroyalblue and Megrey modules were significantly associated with stroke disease. After this step, we interacted DEGs in GSE122709, genes in Meroyalblue and MEgrey modules, and metabolism related gene and showed 554 metabolism related candidate hub genes. Nonetheless, a single WGCNA analysis had significant limitations and inaccuracies (Tzimas et al., 2019). Currently, studies applied WGCNA were normally combined with multiple machine learning algorithms to identify biomarkers for disease prognosis and diagnosis. Zhao et al. (2022) identified four core genes (BTN3A2, CYFIP2, ST8SIA1, and TYMS) as biomarker for diagnosis of rheumatoid arthritis *via* comprehensive analysis of WGCNA, LASSO, random forest, and support vector machine analysis. By WGCNA, LASSO, and random forest algorithms, Fan et al. (2022) obtained five signature genes (UPP1, S100A9, KIF1B, S100A12, SLC26A8) and emerged remarkable diagnostic performance in pediatric septic shock. In the current study, LASSO regression analysis and random forest algorithms found two signature genes, then three validation datasets, including GSE140275, GSE122709, and GSE180470, confirmed that SDHD and FERMT3 were highly expressed in the stroke group.

SDHD, one subunit of succinate dehydrogenase (SDH), dual roles in respiration by transferring electrons from succinate to ubiquinone in the mitochondrial electron transport chain (ETC) and catalyzing oxidation of succinate to fumarate in the mitochondrial Krebs cycle (Cecchini, 2003; Sun et al., 2005). Researchers reported mutations of SDHD in patients with hereditary pheochromocytomas and hereditary paragangliomas (Baysal et al., 2000). *In vitro* experiment performed by Bandara et al. (2021) demonstrated that mutation of SDHD *via* CRISPR/Cas9 approach could suppress glycolysis and overall ATP synthesis in HEK293. Overexpression of SDHD could significantly suppressed cell proliferation *in vitro* and tumor growth of HCC cells *in vivo* (Yuan et al., 2022). FERMT3 is a member of the kindlin family of binding proteins containing the FERM domain (Rognoni et al., 2016). FERMT3 mediates integrin activation and integrin-ligand binding. Therefore, FERMT3 is closely related to various biological activities, including cell adhesion, spreading, cell survival, proliferation and differentiation (Rognoni et al., 2016). Mutations of FERMT3 gene could cause leukocyte adhesion deficiency type III (LAD III) (Kuijpers et al., 2009). Liu et al. (2021) performed RNA sequencing in patients with triple-negative breast cancer and identified FERMT3 as protective gene in compound kushen injection treatment. Nonetheless, correlations of FERMT3 and SDHD with stroke have not been previously reported.

Post-stroke depression (PSD), the most common psychiatric problem after stroke, is an independent risk factor of stroke mortality (D’Anci et al., 2019). PSD is closely associated with worse outcomes of physical and cognitive recovery, functioning, and health related quality of life (Villa et al., 2018). It is worth noting that PSD might halt or impede rehabilitation treatments. However, the complex pathophysiology of PSD is still only partly known till now. The current evidence indicates genetic factors as major aetiopathological predictors for PSD. Yang et al. (2010) reported that IL-18 level in serum on day 7 after admission might predict the risk of PSD. Plasma levels of glutamate and glutamate oxaloacetate transaminase at admission were also reported to be closely related PSD within 3 months (Cheng et al., 2014). To further probe the role of hub genes in stroke, we performed a GSEA analysis of signature genes. The results demonstrated that SDHD and FERMT3 were significantly enriched in depression. Then we validated our findings in stroke patients with and without depression. We found increase expression levels of SDHD and FERMT3 in stroke patients with depression, compatible with our previous research inferences. In addition, based on the two signature genes (SDHD and FERMT3) that we identified, we successfully established a PSD diagnosis for evaluating diagnosis value of SDHD and FERMT3 in our PSD cohort. Nomogram model showed great predictive ability and clinical usefulness. Meanwhile, AUC values of SDHD and FERMT3 were 0.896 and 0.964. Our results suggested that SDHD and FERMT3 might play essential roles in diagnosis of PSD. Finally, we performed correlation analysis of two signature genes and several clinical traits. We found that the SDHD and FERM3 were positively correlated with depression, which suggested that SDHD and FERMT3 had certain therapeutic predictive value in PSD. Moreover, SDHD was also found a negative correlation with activities of daily living in this study. Considering the feature of this parameters, it suggested that these two signature genes may also serve as biomarkers to monitor the mental functional prognosis in patients with PSD (van Hulsteijn et al., 2013).

The present study also has certain shortcomings. Firstly, we collected data from public databases with small samples. There could have been a selection bias. Large datasets of stroke patients are limited, so we tried to minimize the bias of our results by validating signature genes across multiple datasets. Secondly, the metabolism related-pathways and -hub genes in stroke lack literature support and required further confirmation. Thirdly, although two metabolism related signature genes were identified as potential predictors for PSD, larger patient cohorts should be examined in the future to validate the correlation between two signature genes (SDHD and FERMT3) with PSD. Then further *in vivo* or *in vitro* studies should be carried out to validate diagnostic value and potential therapeutic value.

## Conclusion

In conclusion, we identified two signature genes (SDHD and FERMT3) in peripheral blood of stroke patients by machine learning. SDHD and FERMT3 were found to be significantly associated with depression, and were identified as diagnostic and therapeutic signatures by our stroke cohorts with and without PSD, which could be a valuable reference for future clinical practice.

## Data availability statement

The original contributions presented in this study are included in the article/Supplementary material, further inquiries can be directed to the corresponding authors.

## Ethics statement

The studies involving human participants were reviewed and approved by the Committee of Institutional Ethics of the First Affiliated Hospital of Nanjing Medical University. The patients/participants provided their written informed consent to participate in this study.

## Author contributions

YD, YZhe, BY, and RC designed the current study. XZ, XW, and ShuweiW collected the clinical information. YZha and SongW completed data downloading and processing. XZ, ZW, and YZha performed bioinformatics analysis. QY performed ELISA testing. YZhe, XZ, and YZha drafted the manuscript. YD supervised and modified the drafting process. All authors contributed to the article and approved the submitted version.

## Footnotes

1
http://www.ncbi.nlm.nih.gov/geo/

2
https://bioinfogp.cnb.csic.es/tools/venny/index.html

## Supplementary material

Supplementary Table 1 Differentially expressed genes in GSE140275.

Supplementary Table 2 Differentially expressed genes in GSE122709.

Supplementary Table 3 Correlation analysis results of modules and clinical features.

Supplementary Table 4 Signature genes selected by least absolute shrinkage and selection operator (LASSO) analysis.

Supplementary Table 5 Signature genes determined by random forest analysis.
