# Supplementary material for: Machine learning algorithms assisted identification of post-stroke depression associated biological features
Source: Front Neurosci. 2023 Mar 8;17:1146620. doi: 10.3389/fnins.2023.1146620 (PMC10030717; doi:10.3389/fnins.2023.1146620)
Supplement: Supplementary file 1 [file Table_6.docx]

**Supplementary Table 6 Clinical and demographic characteristics of patients with and without post-stroke depression.**

|  | **PSD (n=27)** | **Non-PSD (n=54)** | **P value** |
| --- | --- | --- | --- |
| **Age in year, mean (SD)** | 60.89 (13.67) | 58.33 (13.94) | 0.436 |
| **Gender, n (%)** |  |  | 0.625 |
| **Male** | 16 (59.26) | 35 (64.81) |  |
| **Female** | 11 (40.74) | 19 (35.29) |  |
| **Height in centimeter, mean (SD)** | 168.70 (6.96) | 167.56 (6.64) | 0.472 |
| **Weight in kilogram, mean (SD)** | 69.37(11.14) | 67.04 (12.08) | 0.403 |
| BMI in kg/m2, mean (SD) | 24.29 (3.20) | 23.74 (3.14) | 0.462 |
| **SBP in mmHg, mean (SD)** | 130.07 (14.58) | 125.11 (13.19) | 0.138 |
| **DBP in mmHg, mean (SD)** | 79.52 (9.64) | 77.78 (10.58) | 0.475 |
| **Occupation, n (%)** |  |  | 0.777 |
| **Full-time or part-time** | 13 (48.15) | 20 (37.04) |  |
| **Layoffs** | 0 (0) | 1 (1.85) |  |
| **Retired** | 13 (48.15) | 29 (53.70) |  |
| **Self-employed** | 0 (0) | 1 (1.85) |  |
| **Others** | 1 (3.70) | 3 (5.56) |  |
| **Marriage status, n (%)** |  |  |  |
| **Unmarried** | 0 (0) | 1 (1.85) | 0.739 |
| **Married** | 26 (96.30) | 51 (94.45) |  |
| **Widowed** | 1 (3.70) | 1 (1.85) |  |
| **Divorced** | 0 (0) | 1 (1.85) |  |
| **Others** | 0 (0) | 0 (0) |  |
| **Education, n (%)** |  |  | 0.496 |
| **Primary school or less** | 2 (7.41) | 1 (1.85) |  |
| **Secondary school** | 6 (22.22) | 11 (20.37) |  |
| **High school** | 9 (33.33) | 24 (44.45) |  |
| **College/ University** | 10 (37.04) | 16 (29.63) |  |
| **Postgraduate** | 0 (0) | 2 (3.70) |  |
| **Smoking status, n (%)** |  |  |  |
| **Non-smoker** | 15 (55.55) | 34 (62.96) | 0.421 |
| **Current smoker** | 10 (37.04) | 13 (24.07) |  |
| **Previous smoker** | 2 (7.41) | 7 (12.96) |  |
| **Alcohol intake, n (%)** |  |  |  |
| **No drinking** | 12 (44.44) | 31 (57.41) | 0.527 |
| **Light drinking** | 11 (40.74) | 16 (29.63) |  |
| **Heavy drinking** | 4 (14.82) | 7 (12.96) |  |
| **Medical history, n (%)** |  |  |  |
| **Hypertension** | 14 (51.85) | 32 (59.26) | 0.526 |
| **Diabetes mellitus** | 4 (14.82) | 14 (25.92) | 0.257 |
| **Dyslipidemia** | 4 (14.82) | 6 (11.11) | 0.678 |
| **Coronary heart disease** | 4 (14.82) | 6 (11.11) | 0.678 |
| **HAMD score, mean (SD)** | 14.74 (4.67) | 3.41 (1.58) | <0.001 |
| **NIHSS score, mean (SD)** | 7.33 (6.23) | 9.48 (9.21) | 0.218 |
| BI score, mean (SD) | 41.85 (21.49) | 51.39 (24.81) | 0.093 |
| **mRS score, mean (SD)** | 3.48 (1.05) | 3.70 (1.09) | 0.385 |

PSD: post-stroke depression; SD: standard deviation; BMI: body mass index; SBP: systolic blood pressure; DBP: diastolic blood pressure; HAMD: Hamilton Depression Rating Scale 17-item; NIHSS: National Institute of Health Stroke Scale; BI: Barthel index; mRS: modified Rankin scale.
